# Traditional Japanese herbal medicines for treatment of odontopathy

**DOI:** 10.3389/fphar.2015.00176

**Published:** 2015-08-28

**Authors:** Kojiro Yamaguchi

**Affiliations:** Oral and Maxillofacial Surgery, Kagoshima University HospitalKagoshima, Japan

**Keywords:** Kampo therapy, stomatitis, glossalgia, oral cancer, dry mouth

## Abstract

This article highlights several refractory oral diseases, such as stomatitis, burning mouth syndrome (BMS), glossalgia, atypical facial pain (AFP), oral cancer, dry mouth, and Sjögren's syndrome (SJS), in which use of Japanese herbal medicines, Kampo medicines (KM), on the basis of Kampo theory could exert the maximum effects on human body. (1) In acute stomatitis, heat because of agitated vital energy may affect the head, chest, and middle abdominal region. Stomatitis is also related to the generation of reactive oxygen species (ROS). There are many antioxidants in the crude extracts of KM. Thus, we can control environmental factors (cold, heat, dampness, dryness) and vital energy, blood, and fluid of the organ systemically using KM to treat stomatitis and eliminate local ROS accumulation. (2) BMS, glossalgia, and AFP are multifactorial syndromes involving the interaction of biological and psychological factors. Local temperature decrease and edema often occur in chronic pain. These are local circulatory disturbances that can be resolved by improving the flow of blood and fluid. Several KM, such as Tokishakuyakusan and Kamishoyosan (KSS), are effective for enhancing peripheral circulation. Those such as Saikokaryukotuboreito, Yokukansan, KSS, and Saibokutou can reduce stress and associated pain by altering glutamatergic and monoaminergic transmission in the brain. The clinical efficacy of KM for BMS and AFP may depend on the regulation of the mesolimbic dopaminergic and descending glutamatergic pain modulation systems. (3) Regarding oral cancer treatment, I introduce four possible applications of KM, inhibition of the proliferation of cancer cells, complementation of the main cancer therapy, reduction of side effect caused by the main anti-cancer therapy and improvement of quality of life such as the overall status and/or oral discomfort. This review explains in more details Hozai such as Hochuekkito (HET), Juzendaihoto, and Ninjinyoeito (NYT) that are frequently used to improve both immunosuppression and deficiencies of Ki, Ketsu, and Sui in oral cancer patients. (4) Heat- and cold-dryness stages exist in dry mouth and SJS. Byakkokaninjinto is useful for heat-dryness, while NYT, Bakumondoto, and HET have moisturizing effects in the cold-dryness stage. Thus, Kampo therapy is useful for many oral diseases that cannot be cured by western medicine.

## Introduction

The human oral cavity is an anatomically complex structure that has evolved to perform a multitude of functions, including mastication, swallowing, tasting, and articulation. The oral cavity is also the initial digestive organ, and thus critical for nutrition and gastrointestinal function. These functions require an extensive and highly integrated system of sensorimotor control pathways. Thus, oral sensation and function are sensitive to local stimuli, oral disease, general health status, systemic disease, resident microorganisms, and even psychological and emotional states. Systems of traditional medicine treat oral discomfort and odontopathy not only by considering anatomical and intraoral environmental factors (i.e., cold, heat, dampness, and dryness), but from a holistic perspective considering general physical and psychological health. Kampo theory, a branch of traditional Japanese medicine emphasizing the treatment of vital energy (Ki), blood, and fluid abnormalities using herbal preparations, has proven to be highly effective for the treatment of oral discomfort, resulting from various diseases. The Japanese traditional herbal formulae, known as Kampo, have gradually re-emerged, and approximately 20–30 different formulations (mainly herbal extracts) can be prescribed for treatment of odontopathy.

The objective of this article is to introduce and summarize pharmacological actions and clinical applications of Japanese herbal medicines in various refractory oral diseases such as stomatitis (Section Stomatitis), burning mouth syndrome (BMS), glossalgia (glossodynia), atypical facial pain (AFP) [Section Burning Mouth Syndrome (BMS), Glossalgia (Glossodynia), and Atypical Facial Pain (AFP)], oral cancer (Section Oral Cancer), dry mouth, and Sjögren's syndrome (SJS) (Section Dry Mouth and Sjögren's Syndrome). The literature search used Evidence Reports of Kampo Treatment 2010: 345 Randomized Controlled Trials (EKAT 2010) (Okabe and Tsutani, 2010) and EBM of Kampo Medicine in Oral Surgery (2015) (Wang et al., 2015). Furthermore, Pubmed and ICHUSHI [Japan Medical Abstracts Society (Jamas)] were used as search engines. This article includes 63 reports.

Ijima et al. reported the clinical usefulness of Kampo for the treatment of intractable oral diseases such as dry mouth, glossodynia, feeding and swallowing disorders, and intractable stomatitis in 35 medically compromised patients (5 male and 30 female) (Ijima et al., 2007).

Kampo medicines (KM) controlled 20 cases, with complete alleviation of symptoms (57.1%), while 14 cases showed improvement (40.0%). Overall, Kampo treatment was extremely useful in 28 cases (80.0%) and considerably useful in three cases (8.6%).

Ara et al. conducted a series of experiments testing the efficacy of Kampo herbal medicines on periodontal disease, drug-induced gingival overgrowth, and xerostomia (dry mouth) using *in vitro* cultures and animal models. Shosaikoto (SST), a formula containing a variety of bioactive flavones, saponins, and gingerols, decreased lipopolysaccharide (LPS)-induced prostaglandin E2 (PGE2) production by gingival fibroblasts; this is consistent with anti-inflammatory activity in periodontal tissues. In addition, Saireito inhibited the proliferation of gingival fibroblasts in response to the calcium channel blocker nifedipine (among several drug classes associated with gingival hyperplasia, including anticonvulsants) and was effective against idiopathic retroperitoneal fibrosis (Ara et al., 2010). In addition, both the Byakkokaninjinto (BNT) and Goreisan (GRS) formulae enhanced saliva secretion to mitigate xerostomia, which is commonly associated with diabetes, in the streptozotocin-induced diabetic mouse model. Horie et al. demonstrated that Rikkosan (RKS) reduced PGE2 by selectively inhibiting cyclooxygenase-2 (COX-2) activity in LPS-stimulated mouse macrophage-like RAW264.7 cells (Horie et al., 2008).

Oral candidiasis (thrush), which often occurs in denture wearers and people with systemic immunosuppression, manifests with similar symptoms (such as oral bitterness, glossalgia, stomatitis-like symptoms, and oral dryness).

Then the treatment of oral candidiasis is usually used anti-fungal medicines, therefore, it should be ruled out beforehand.

If those symptoms remain even that have treated candidasis, they are candidates for Kampo therapy.

## Stomatitis

Kampo formulae are effective for treating oral diseases such as stomatitis and oral lichen planus (Zheng et al., 2011). Stomatitis presents with inflammatory manifestations including reddening, erosion, and ulceration of the oral mucosa. Stomatitis may occasionally be multiple or recurrent. In Kampo medicine, stomatitis treatment differs depending on whether the symptoms are intense acute or chronic. In the acute type, heat due to agitated vital energy may affect the head, chest, and middle abdominal region. Therefore, treatment is required to cool the heat in the heart, stomach, and liver using formulae with cooling effects such as Hangeshashinto (HST), Orento (ORT), Orengedokuto (OGT), or BNT.

In the chronic type, prolonged head, chest, and middle abdominal heat causes fluid deficiency. Therefore, formulae are required not only to alleviate the heat but also to treat vital energy and blood, and to increase wetness by increasing fluid retention. In this case, the following formulations are often selected for specific symptoms: (1) middle abdominal region deficiency-treating formulae such as the Kenchuto group; (2) Rokumigan and Hachimijiogan; (3) vital energy deficiency formulae such as Hochuekkito (HET) and Rikkunshito (RKT); (4) dual deficiency-treating formulae such as Juzentaihoto (JTT) and Ninjinyoeito (NYT); and (5) other formulations such as Jiinkokato, Unseiin, and Tokishakuyakusan (TSS).

Kampo formulae may also suppress the underlying causes of stomatitis, particularly infection, inflammation, and concomitant oxidative stress and drug induced type.

Scutellariae Radix, Coptidis Rhizoma, Cinnamomi Cortex, Glycyrrhizae Radix, Astragali Radix, and Phellodendri Cortex inhibited several bacterial infection.

Scutellariae Radix, Cinnamomi Cortex, Anemarrhenae Rhizome, and Menthae Herba had antifungal effect. These crude drugs were included in HST, ORT, NYT, HET, JTT, BNT, and Kamishoyosan (KSS).

And Glycyrrhizae Radix and Scutellariae Radix inhibited viral infection (Toriizuka, 2003).

Li et al. found that procyanidin B1 purified from Cinnamomi cortex inhibited infection by vesicular stomatitis virus and suppresses hepatitis C virus replication (Li et al., 2010).

Astragali Radix, Scutellariae Radix, Phellodendri Cortex, Coptidis Rhizoma, Glycyrrhizae Radix, Platycodi Radix, Bupleuri Radix, Paeoniae Radix, Atractylodis Lanceae Rhizoma, Cimicifugae Rhizoma, Cnidii Rhizoma, Angelicae Radix, Poria, Processi Aconiti Radix, and Ephedrae Herba had anti-inflammatory effects.

And Paeoniae Radix, Cimicifugae Rhizoma, Cnidii Rhizoma, Angelicae Radix, and Processi Aconiti Radix had anodyne effect (Toriizuka, 2003).

These crude drugs were included in HST, ORT, NYT, HET, JTT, Kikyoto, KSS, TSS, and Keisikajutubuto (KJT).

Many of these formulae are rich in antioxidants. Dragland et al. (2003) assessed the contribution of culinary and medicinal herbs to the total dietary intake of antioxidants.

There are few effective treatment options for drug-induced stomatitis, such as that associated with cancer chemotherapy. However, HST has a preventive effect on stomatitis associated with radiation therapy and chemotherapy.

Kono et al. reported that 13 of 14 patients (92.8%) afflicted with chemotherapy-induced oral mucositis (COM) during mFOLFOX6 or FOLFIRI treatment for metastasis of advanced colorectal cancer showed significant improvement following a 1-week topical treatment (3 times daily oral rinse) with HST, with significantly decreased mean Common Terminology Criteria for Adverse Events (CTCAE) grade (*P* = 0.0012) (Kono et al., 2010). Aoyama et al. conducted a double-blind, placebo-controlled, randomized study of HST for COM from gastric cancer treatment. Although HST treatment did not reduce the incidence of grade 2 or higher COM, a trend was observed in which HST reduced the risk of grade 1 COM during the screening cycle (Aoyama et al., 2014).

OGT significantly improves mucositis caused by anticancer agents (Okumi and Koyama, 2014).

These studies suggest that the expression of stomatitis is related to generation of reactive oxygen species (ROS) and that antioxidants contained in medicinal herbs can effectively mitigate this damage (Oka, 1995; Dragland et al., 2003; Horie et al., 2008; Yamaguchi et al., 2008; Kono et al., 2010; Li et al., 2010; Zheng et al., 2011; Aoyama et al., 2014). A list of antioxidants in Chinese and Japanese medicinal herbs and products is presented in Table 1. Antioxidant scores are from Dragland et al. (2003).

**Table 1:** **Antioxidants in Kampo medicine and crude drugs**.

Crude drug (Antoxidant mmol/100 g)^*^	**Kampo medicine**						
	**Oren To (ORT)**	**Hange Shasin To (HST)**	**Sho Saiko To (SST)**	**Ninjin Youei To (NYT)**	**Hochu Ekki To (HET)**	**Touki Shakuyaku San (TSS)**	**Byakoka Ninjin To (BNT)**
Glycyrrhiae Radix (11.6)^*^	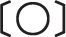	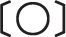	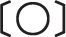	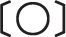	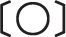		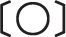
Ginseng Radix (1.5)^*^	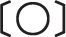	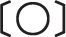	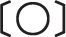	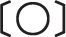	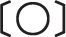		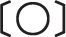
Zizyphi Fructus (5.9)^*^	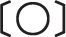	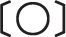	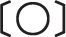		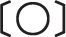		
Angelicae Radix (3.0)^*^				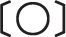	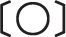	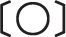	
Pinelliae Tuber (0.3)^*^	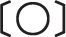	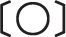	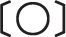				
Cinnamomi Cortex (120.2)^*^	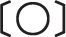			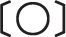			
Scutellariae Radix (111.5)^*^		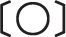	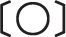				
Paeoniae Radix (55.1)^*^				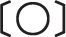		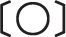	
Zingberis Rhizoma (7.5)^*^			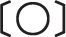		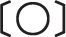		
Atractylodis Lanceae Rhizoma (7.4)^*^				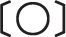	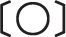	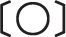	
Bupleuri Radix (5.7)^*^			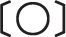		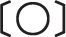		
Astragali Radix (4.9)^*^				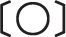	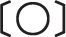		
Poria (Holelen) (2.8)^*^				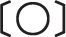		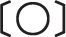	
Cimicifugae Rhizoma (64.3)^*^					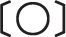		
Aurantii nobilis Pericarpium (17.5)^*^				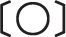			
Cnidii Rhizoma (6.7)^*^						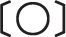	
Rhemanniae Radix (3.9)^*^				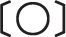			

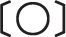
 *Indicate the contents of Kampo medicine.*

* Antoxidant value (mmol/100 g) of crude drug.

The Chinese and Japanese medicinal herbs Cinamomi Cortex, Scutellariae Radix, and Paeoniae Radix contained >50 mmol antioxidants per 100 g, while Saikokeishito, JTT, and HET contained 9.7–21.4 mmol antioxidants per 100 g (Dragland et al., 2003). Oka et al. conducted a randomized controlled trial on the efficacy and safety of ORT for the treatment of 30 acute aphthous stomatitis cases, and found faster resolution of pain in the ORT extract group (4.5 g t.i.d.) (Oka, 1995). Yamaguchi et al. described four cases of aphthous stomatitis treated with Kampo medicine, three of which were treated with NYT, HET, and/or TSS because of vital energy deficiency and/or blood circulatory function deficiency, while the other case was treated with BNT to mitigate an internal hot state associated with oral candidiasis. Two or three months later, all cases were completely healed (Yamaguchi et al., 2008).

## Burning mouth syndrome (BMS), glossalgia (Glossodynia), and atypical facial pain (AFP)

BMS is characterized by chronic pain in the tongue or other oral mucous membranes in the absence of any visible abnormality or organic disease (Baker and Savage, 2005; Klasser et al., 2008, 2011; Ferensztajn et al., 2013). Burning mouth patients typically exhibit pain symptoms bilaterally in the mouth, with the most commonly affected sites being the tongue and lip. Glossalgia (glossodynia) is a disease in which tongue pain occurs without abnormal findings in the lingual mucosa. Characteristic features of glossalgia are as follows. (1) It is common in women at a cancer-prone age (middle-aged and older). Menopausal or postmenopausal hormone changes, stress, anxiety, and nervousness are involved in aggravation and continuation of symptoms. (2) It occurs more frequently in the tip and lateral margins of the tongue but infrequently at the back of the tongue. (3) The pain mitigates or resolves on talking or eating (Yamaguchi, 2013). Both AFP and atypical dental pain refer to chronic persistent pain in the oral cavity, jaw, face, and/or teeth without identifiable organic causes. However, these symptoms may be related to somatoform and psychological factors because it is generally accepted that the pain is independent of peripheral nerve activity (Yamaguchi, 2013).

In the International Classification of Headache Disorders II, the International Headache Society categorizes BMS as cranial neuralgias (International Headache Society, 2004). The etiology of BMS is currently unknown; however, most studies support a multifactorial syndrome involving the interaction of biological and psychological factors (particularly paresthesia, altered taste sensation, blood stasis, and mouth dryness due to the lack of fluid, and depression) (Baker and Savage, 2005; Klasser et al., 2008, 2011; Ferensztajn et al., 2013; Yamaguchi, 2013).

### Nerve system related with BMS

#### BMS-associated changes in peripheral nerve function

Lauria et al. investigated the role of trigeminal small-fiber sensory neuropathy in BMS. Patients showed a significantly lower density of epithelial nerve fibers than controls and remaining fibers were diffuse, reflecting axonal degeneration (Lauria et al., 2005). Yilmaz et al. investigated the contribution of the capsaicin receptor (transeint receptor potential vanilloid receptor-1:TRPV1), which mediates heat-like oral pain, and nerve growth factor (NGF) in BMS. There were fewer nerve fibers penetrating the epithelium (*p* < 0.0001), again consistent with small fiber neuropathy, while there was a significant increase in TRPV1-positive fibers (*p* = 0.0011). Moreover, there was a significant correlation between the baseline pain score and expression of both TRPV1-positive (*p* = 0.0143) and NGF-positive fibers (*p* = 0.0252), and patients were more sensitive than controls to capsaicin-induced pain. Thus, selective TRPV1 and NGF blockers may provide a new therapy for BMS (Yilmaz et al., 2007).

#### Central pain mechanisms of BMS

Capsaicin-induced TRPV1 receptor-mediated pain can induce a distal analgesic effect (pain-induced analgesia) as evidenced by elevated paw mechanical withdrawal threshold in rats (Gear et al., 1999). This pain-induced analgesia was blocked by pre-injection of a dopamine antagonist into the nucleus accumbens, implicating the Central nerve system (CNS) in the modulation of BMS-associated pain (Gear et al., 1999). Recently, Jääskeläinen et al. and Hagelberg et al. reported changes in putamenal/striatal dopamine receptor expression in patients with BMS or AFP using positron emission tomography (PET) imaging (Jääskeläinen et al., 2001; Hagelberg et al., 2003a,b, 2004). Increased binding of the D2 antagonist ^11^C-raclopride and a concomitant decrease in the D1/D2 ratio may indicate a decline in endogenous dopamine levels in the putamen of BMS patients, suggesting that dysfunction of dopamine-mediated central pain inhibition contributes to BMS (Hagelberg et al., 2003b). Furthermore, Hagelberg et al. reported a similar increase in D2 receptor availability and decrease in D1/D2 ratio in the putamen of patients with AFP, again suggesting that the nigrostriatal dopaminergic system may be impaired in chronic orofacial pain (Hagelberg et al., 2003a, 2004). In light of these findings, the effects of several Kampo formulations, including Saikokaryukotsuboreito (SRB), Yokukansan (YKS), KSS, and Saibokuto (SBT), on the mesolimbic system have been examined for efficacy against these orofacial pain disorders. These formulae are broadly effective treatments for BMS, glossalgia, atypical maxillofacial pain, and atypical dental pain (Yamaguchi, 2013) as discussed below. Moreover, these agents appear to suppress the psychological sequel of depression and anxiety.

#### Effective kampo medicine for psychogenic BMS

##### Saikokaryukotsuboreito (SRB)

Sasaki et al. found that preadministration of SRB (600 mg/kg, p.o.) suppressed the increase in amygdalar, hypothalamic, and thalamic dopamine, and 3,4-dihydroxy-phenyl acetic acid (DOPAC) in mice exposed to psychological stress and conditioned fear, but not in mice subjected to electric shocks (Sasaki et al., 1998). In addition, Mizoguchi et al. reported that SRB ameliorated the stress-induced depressive state and reversed the decrease in both extracellular serotonin and dopamine in the prefrontal cortex (PFC). Thus, SRB may ameliorate the chronic stress-induced depressive state by normalizing both cortical and striatal dopamine (Mizoguchi et al., 2010).

##### Yokukansan (YKS)

Mizoguchi et al. reported anxiolytic effects of YKS in aged rats as evidenced by increased open arm activity in the elevated plus maze, and found that YKS reversed the age-related decreases in extracellular dopamine and serotonin within the aged PFC (Mizoguchi et al., 2010).

Suzuki et al. reported suppression of mechanical and thermal allodynia by YKS in the chronic constriction injury model of chronic pain. In addition, YKS significantly reduced the increase in cerebrospinal fluid glutamate induced by mechanical or cold stimuli, while glutamate transporter inhibitors (DL-threo-beta-hydroxy aspartate, and dihydrokainate) suppressed these antiallodynic effects. Thus, YKS-induced antiallodynia in rats appears to be mediated by reduced spinal glutamatergic neurotransmission via enhanced glutamate transporter activity (Suzuki et al., 2012). These reports (Sasaki et al., 1998; Mizoguchi et al., 2010; Suzuki et al., 2012) indicate that herbal medicines can reduce stress and associated pain by altering glutamatergic and monoaminergic transmission in hypothalamus, amygdala, PFC, and spinal cord. The clinical efficacy of KM for BMS and AFP may depend on regulation of the mesolimbic dopaminergic and descending glutamatergic pain modulation systems.

##### Saibokuto (SBT)

SBT is effective for glossalgia (including laryngopharyngeal discomfort) associated with fluid retention in the ShoYo stage (the stage of disease transformation with heat halfway between exterior and interior, characterized by bitter taste). Bessho et al. compared the efficacy of SBT to tranquilizer plus vitamin B complex combination therapy for patients with glossodynia. Each of the subjective symptoms (pain, burning sensation, and unpleasant feeling) was evaluated on a 10-point scale. It was found that SBT reduced pain and burning sensation in glossodynia patients more effectively than diazepam plus vitamin B complex therapy. The majority of SBT-treated patients (92%) reported good or excellent responses after 3 months compared to only 69% in the diazepam group (Bessho et al., 1998).

##### St. John's wort

It is also said that St. John's wort is effective for depression. Klasser studied the herbal supplement Hypericum perforatum (St. John's wort) for management of BMS as one of the systemic medications (Klasser et al., 2011).

### Cold/heat with microcirculation disorder

For effective Kampo treatment, it is important to determine whether the pain is due to cold/heat imbalance or to stagnation of Ki, blood, and/or fluid. Chen et al. reported 18 cases of glossodynia exhibiting a microcirculation disorder of the tongue (Chen et al., 2000). Glossalgia in patients with a deficiency pattern associated with vital energy depression can be treated with HET, which improves quality of life and immunological status of weakened postoperative patients by upregulating the microcirculation.

Another treatment option is TSS, which is used for glossalgia associated with general malaise, fatigue proneness, menstrual disorder, palpitation, or autonomic nerve imbalance, particularly in patients with “white fur tongue” and dark-red punctate color change on the lingual tip and margins. KSS is used for nonspecific complaints and upper heat/lower cold in deficiency-pattern patients. Redness at the tip of the tongue indicates upper-body heat and is a good indication for this formula. Both TSS and KSS improve the microcirculation.

Takayama et al. reported that TSS increases ocular blood flow (OBF) in healthy subjects compared with control (*p* < 0.01) and from 30 to 60 min after administration compared with baseline (*p* < 0.05) (Takayama et al., 2014). Goto examined the efficacy of Kampo for blood stasis syndrome in Japan. There are many signs of this syndrome, such as a dry mouth, fullness of the abdomen, and rough skin. Patients with blood stasis syndrome showed hemorheological abnormalities, and an improvement in these abnormalities was found after administration of removing-blood stasis formulae. Blood stasis syndrome is more than just a circulatory deficiency; it encompasses the pathological concept of constant multilateral change in the living body (Goto, 2014).

Yamaguchi et al. reported that HET, TSS, and/or KSS reduced visual analog scale (VAS) pain score by 92.4% after 19.4 weeks in BMS patients (Yamaguchi et al., 2007). In addition, Yamaguchi et al. reported a case of BMS treated with BNT, which reduced the VAS score from 44 mm to almost 0 after 18 weeks (Yamaguchi et al., 2005). Thus, BNT is appropriate for patients with an interior heat pattern associated with oral dryness; therefore, it is effective, particularly for glossalgia associated with nocturnal thirst. Kikyoto (KKT) is also effective as an anti-inflammatory and heat pattern-treating (Seinetsu) formulation. Yamaguchi et al. reported a case of glossodynia with erythrokeratoderma treated with both internal and gargled KKT. After 6 weeks, the VAS scale of glossodynia was reduced from 47 to 11 and skin symptoms were also relieved. By 11 weeks of treatment, glossodynia VAS was reduced by 85% and skin symptom VAS by 43% (Yamaguchi et al., 2009).

### Kampo theory and herbal constituents

Kampo theory encompasses Yin/Yo theory, Ki (vital energy), Ketsu (blood), and Sui (fluid) theory, and five viscera theory.

#### Yin/Yo theory

The Yin/Yo theory is based on the philosophical concept of well-balanced pairs of complementary aspects, such as heaven and earth or day and night. Medical, excess, heat, and Ki belong to the Yo category, while deficiency, cold, blood, and fluid (visible circulating element) belong to the Yin category (The Japan Society for Oriental Medicine, 2005).

#### Ki·Ketsu·Sui theory

Ki is invisible circulating vital energy and belongs to the Yo category. Ki disturbance is manifested by regurgitation of Ki and depression of Ki. Ketsu indicates blood (one of the visible circulating elements) and belongs to the Yin category.

Ketsu disturbance is manifested by blood stasis (Oketsu) and Ketsu deficiency.

Sui is fluid (one of the visible circulating elements) that belongs to the Yin category. Sui disturbance is manifested by accumulation of Sui and impaired water excretion (The Japan Society for Oriental Medicine, 2005).

#### Five parenchymatous viscera theory

Five parenchymatous viscera (liver, heart, spleen, lung, and kidney) are used as functional units in Kampo theory. The concept of five parenchymatous viscera encompasses not only the viscera of western medicine but also several differential functional units. Every viscera produces Yin and Yo products. In the abnormal stage, the deficiency or excess in Yin and/or Yo, or a dual deficiency is observed in the body (The Japan Society for Oriental Medicine, 2005).

Hijikata et al. recommended application of Kampo theory to determine the appropriate herbal prescriptions for patients with incurable glossodynia (Hijikata et al., 2008). KM used for patients with incurable glossodynia are as follows: RKT, Jiinkokato, Hachimijiogan, Ryutanshakanto, Seishoekkito, Bakumondoto (BMT), Kamikihito, Chikujountanto, Rokumijiougan, KSS, and Kambakutaisoto. KM, properly prescribed on the basis of Kampo theory, quickly resolved the pain of refractory glossodynia (Yamaguchi et al., 2009). Okamoto et al. reported that 69.2% of glossodynia patients treated by Kampo responded well, with an average delay to improvement of 8.0 ± 7.7 weeks. The most common herbs in the aforementioned preparations for glossodynia are Glycyrrhiza Root, Ginseng Root, Poria (Holelen), and Atrctylodes (lancea) Rhizome, which together comprise an essential Kampo prescription called Shikunshito. The two most frequently used KM for glossodynia in patients showing high response (reduction in severity of 50% or more) are Seinetsuhokito and Mibakuekkito (Okamoto et al., 2003).

A summary of KM used for the treatment of BMS, glossalgia, and AFP is presented in Table 2 (Bessho et al., 1998; Sasaki et al., 1998; Okamoto et al., 2003; The Japan Society for Oriental Medicine, 2005; Yamaguchi et al., 2005, 2007, 2009; Hijikata et al., 2008; Mizoguchi et al., 2010; Suzuki et al., 2012; Yamaguchi, 2013). The most effective constituent crude drugs are Glycyrrhiae Radix, Atractylodis Lanceae Rhizoma, Angelicae Radix, Bupleuri Radix, Ginseng Radix, Zingberis Rhizoma, and Poria (Holelen) (Bessho et al., 1998; Sasaki et al., 1998; Okamoto et al., 2003; The Japan Society for Oriental Medicine, 2005; Yamaguchi et al., 2005, 2007, 2009; Hijikata et al., 2008; Mizoguchi et al., 2010; Suzuki et al., 2012; Yamaguchi, 2013) (Table 3).

**Table 2:** **Kampo medicines for BMS, glossalgia, and AFP**.

**Target and Kampo medicine**	**Effect**
**1) Nervous system** Saikokaryukotuboreito	Ameliorates chronic stress-induced depressive state by preventing prefrontal cortex dysfunction
**1) Nervous system** Yokukansan	Antiallodynic effects in rats related to blockade of spinal cord glutamatergic neurotransmission via activation of glutamate transporters
**1) Nervous system** Saibokuto	Improves glossodynia more effectively than diazepam plus vitamin B complex
**2) Hot/cold, microcirculation** Hochuekkito	Improves symptoms of BMS by correcting “Ki deficiency”
**2) Hot/cold, microcirculation** Tokishakuyakusan	Improves the symptoms of BMS by correcting deficiency of “Ketsu” and microcirculation
**2) Hot/cold, microcirculation** kamishouyousan	Improves “Yo” by correcting ”kidney Yin” and “liver Yin” deficiency, as well as restless organ disorder based on Yin deficiency of five viscera
**2) Hot/cold, microcirculation** Byakko-ka-ninjinto	Improves BMS symptoms such as dry mouth and intraoral pain
**2) Hot/cold microcirculation** Kikyoto	Improves glossodynia and erythrokeratoderma
**3) Kampo theory** Rikkunshito	Improves “spleen vital energy” and “heart Yin” deficiency, stagnation of “liver Ki (vital energy) producing fire,” and “gallbladder Ki (vital energy)” deficiency
**3) Kampo theory** Jiinkokato, Hachimijiogan, Ryutanshakanto	Improves “spleen Yin” and “heart Yin” deficiency, “kidney Yo” deficiency, and “liver Ki (vital energy)” stagnation producing heart syndrome
**3) Kampo theory** Seishoekkito, Bakumondoto	Improves “spleen Ki (vital energy) “ deficiency and “stomach Yin” deficiency producing heart syndrome
**3) Kampo theory** Bakumondoto	Improves “spleen Ki” deficiency and “stomach Yin” deficiency producing heart syndrome
**3) Kampo theory** Kamikihito, Chikujountanto	Improves “spleen Ki” and “heart Yin” deficiency, stagnation of “liver Ki producing fire,” and “gallbladder Ki” deficiency
**3) Kampo theory** Rokumijiougan, Kanbakutaisoto	Improves Yo rise based on “kidney Yin” and “liver Yin” deficiency as well as restless organ disorder based on Yin deficiency of five viscera
**3) Kampo theory** Hangeshashinto, Anchusan	Improves “spleen Yo” deficiency
**3) Kampo theory** Mibakuekkito, Seinetsuhokito	Improves glossodynia

**Table 3:** **Frequently used Kampo medicines and the effective herbs for BMS, glossalgia, and atypical orofacial pain**.

**Effective crude drugs**	**Medicines**						
	**Seinetu hokito**	**Saibokuto**	**Kami Shoyosan**	**Mibaku Ekki To**	**Hochu Ekki To**	**Toki Shakuyaku San**	**Yoku kan san**
Glycyrrhiae Radix	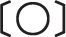	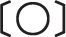	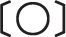	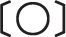	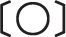		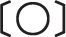
Atractylodis Lanceae Rhizoma	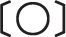		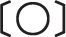	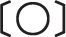	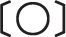	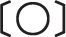	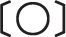
Angelicae Radix	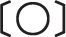		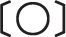	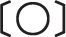	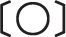	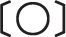	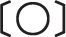
Bupleuri Radix		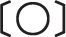	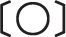	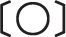	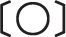		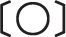
Ginseng Radix	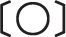	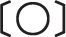		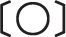	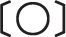		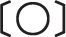
Zingberis Rhizoma		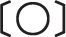	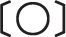	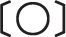	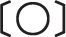		
Poria (Holelen)	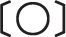		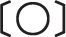			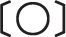	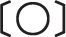
Cimicifugae Rhizoma	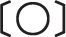			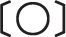	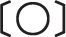		
Paeoniae Radix	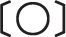		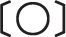			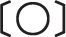	
Zizyphi Fructus				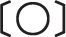	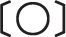		
Astragali Radix				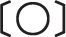	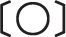		
Pinelliae Tuber		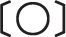					

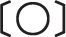
 *Indicate the contents of Kampo medicine.*

## Oral cancer

The surgical remedy, chemotherapy, and radiotherapy are main treatment strategy of oral cancer. From the standpoint of providing mild therapy in cancer treatment, herbs and herbal combinations have attracted a great deal of attention. In conjunction with this phenomenon, the actions of many herbs are now being examined at the pharmacologic and molecular levels, suggesting that increased scientific understanding of the actions of many traditional herbal remedies may identify compounds with novel therapeutic uses. Several researchers and oncologists have reported the direct inhibitory effects of herbs or herbal combinations on the proliferation of cancer cells (Liao et al., 2005; Yang et al., 2011). The main purpose of additive kampo therapy in patients with oral cancer is to complement the main anti-cancer therapy, to reduce side effect caused by the main anti-cancer therapy, and to improve quality of life such as the overall status and/or oral discomfort. Taken together, I here introduce four possible applications of KM to the treatment of oral cancer.

### Direct inhibitory effects of herbs or herbal combinations on the proliferation of cancer cells

Liao et al. reported the induction of apoptosis in human oral cancer cell lines by Chingwaysan (CWS), a Chinese herbal medicine containing Cimicfugae Rhizoma, Rehmanniae Radixet Rhizoma, Moutan Radicis Cortex, Coptidis Rhizoma, and Angelicae Sinensis Radix, well known as an effective treatment for intraoral ulcerative lesions and gingival bleeding. CWS inhibited the proliferation of human OC2 and TSCCa cells, accompanied by morphological changes, reduced cell viability, and internucleosomal DNA fragmentation consistent with apoptotic cell death. Furthermore, CWS induced a dose-dependent increase in the proapoptotic Bax protein (Liao et al., 2005).

Salvia miltiorrhiza (Danshen) has been used for treating angina pectoris, myocardial infarction, and for improving blood flow. Ya Yang et al. reported that salvianolic acid B (Sal B) inhibited proliferation of two oral squamous cell carcinoma (OSCC) cell lines, CAL27 and SCC4. Moreover, DNA microarray analysis of genes involved in angiogenesis revealed 17 showing a greater than three-fold change in expression. Among these, hypoxia inducing factor-1α (HIF-1α), tumor necrosis fuctor- α (TNFα), and matrix metalloproteinase-9 (MMP9) were inhibited and expression of thrombospondin-2 (THBS2) was upregulated (Yang et al., 2011).

### Complementation of the main anti-cancer therapy

Satoh et al. found that HET increased lymphocyte cell-surface antigens, CD3-positive cells, and CD3/CD4 double-positive cells (*p* < 0.05) in elderly patients with chronic weakness, and this increased immune function was associated with increased QOL (Satoh et al., 2005).

Ikemoto et al. showed the immunoaugumentation effects of JTT empirically considered an immunoaugumentation drug, by investigating regulatory T cell (Treg) levels and other immunological parameters in 30 patients with advanced pancreatic cancer. Peripheral Foxp3+ Treg populations, CD4/CD8 ratio, and CD57+ cell (natural killer, NK cell) populations were estimated after 14 days of JTT administration prior to anticancer therapy. Administration of JTT significantly decreased Treg populations (*P* < 0.001) and increased the CD4/CD8 ratio (*P* < 0.01), while CD57+ cell populations did not significantly change (Ikemoto et al., 2014).

Matsuda et al. reported that oral administration of JTT increases the production of Interleukin-12 (IL-12) in the lungs, which is responsible for the activation of both NK and NKT cells, resulting in inhibition of B16 melanoma cell metastasis (Matsuda et al., 2011).

### Reduction of side effect caused by the main anti-cancer therapy

JTT include the crude drugs (Angelicae Radix, Paeoniae Radix, Cnidii Rhizoma, and Rehmanniae Radix) with the hematopoiesis. Therefore, JTT is used for anemia and leukocytopenia as an adjuvant therapy.

Ogawa et al. evaluated the efficacy of JTT on alleviating myelosuppression induced by TS-1 therapy in mice, and explored biomarkers that reflect both bone marrow suppression by TS-1 and alleviation by JTT using a proteomics approach. After administration of TS-1, a significant decrease in White blood cell (WBC) count and CD34+ bone marrow cells (BMC) ratio were observed on days 5 and 3, respectively. JTT treatment improved WBC count on day 7 and CD34+ BMC ratio on days 5 and 7. Surface-enhanced laser desorption analysis identified three protein peaks that increased on day 3 after treatment with TS-1 but remained unchanged in mice cotreated with JTT (Ogawa et al., 2012). This study indicates that bone marrow suppression by TS-1 treatment in mice may be improved by coadministration of JTT. A C-terminal fragment of albumin was identified as a candidate biomarker for predicting TS-1-induced myelosuppression.

Ohara et al. evaluated the clinical effects of HET and NYT in patients with gastric cancer, colorectal cancer, breast cancer, and other cancers undergoing tegafur chemotherapy in a randomized controlled trial. In that study, loss of appetite was significantly improved by HET, while nausea/vomiting, bowel movement abnormalities, motivation, and fatigue/malaise were significantly improved by NYT. Overall improvement was 36.8% for HET and 33.9% for NYT compared to only 14.3% for tegafur alone (Ohara et al., 1993).

To control cancer pain that can result from the cancer itself, complementation of KM is essential. Yamaguchi et al. (2002) reported a case of tongue cancer with touch pain at meal time successfully treated with RKS, NYT, and BNT. In that report (Yamaguchi et al., 2002), Pain was well controlled and the general condition improved during cancer treatment. RKS contains several herbal essences that have local anesthetic and analgesic effects. RKS was gargled 10 min before meals and was effective for 30–40 min after rinsing. NYT proved useful for relaxation, while BNT was used to reduce the symptoms of radiostomatitis and xerostomia induced by radiotherapy. It is also known that local temperature control (Cold, Hot) is important for pain control (Yamaguchi et al., 2002). Yamaguchi et al. reported the case of a 42-year-old male with chronic neuralgia-like pain, following cyber knife therapy to treat recurrent mandibular malignant fibrous histiocytoma, successfully treated with KJT. In that report, VAS for pain decreased by 20% after 2 weeks of treatment and was abolished after 4 weeks, with no reported recurrence (Yamaguchi et al., 2011). Pain control was associated with improved bilateral surface skin temperature.

### Improve quality of life such as the overall status and/or oral discomfort

Takita et al. observed spontaneous regression of tongue cancer in a 72-year-old terminal patient. JTT treatment (5 g/day) was started without anticancer therapy, first orally and then by tube feeding. Starvation gradually progressed. Tumor volumes both in the oral cavity and neck gradually decreased, and bleeding and pain were almost absent up to the terminal endpoint (total inpatient period: 171 days). Moreover, the tumor had almost disappeared on CT images acquired 5 h postmortem. The progression to a comfortable natural death may have been aided by spontaneous regression of the tumor (Takita et al., 2011).

Misumi et al. reported clinical effects of JTT in 16 oral cancer patients. In the 10 group A members in whom treatment was not indicated because of severity, palliative care was performed. The course was frequently stable, with reduced cancer pain, and seven of these patients did not require opioids. In contrast, in the 6-group B members with multiple primaries or oral cavity cancer, localized treatment was applicable, and the course was favorable (Misumi et al., 2013).

JTT is an effective ancillary agent for those with age-, lifestyle-, or treatment-related immunodeficiency due to oral cancer.

JJT inhibits the progression of liver tumors in a dose-dependent manner and contributes to long-term survival, and RKT contributes to the amelioration of anorectic conditions in cancer cachexia-anorexia syndrome (Okumi and Koyama, 2014).

Yamaguchi et al. reported oral discomfort associated with cancer treatment that were successfully treated with NYT. The first was a 70-year-old female who complained of long-term tongue pain at the right edge and multiple stomatitis. She had a history of rectal cancer surgery at 33 and 39 years old, and was hospitalized at age 69 for bowel dysfunction. Clinical diagnoses were Candidal stomatitis and dry mouth. NYT 6 g/day was applied, and an antifungal drug was used for gargling; symptoms were almost absent after 4 months (Yamaguchi et al., 2012). A 68-year-old male complained of xerostomia and multiple stomatitis who had a history of prostate cancer surgery at 61 years and lung cancer treatment at 62 and 63 years. Before Kampo treatment, erosion of the mouth wing and dry tongue were noted, and the Saxon test for saliva production yielded only 0.6 g/2 min. NYT 9 g/day was applied and KKT gurgled. Two weeks later, parotid saliva flow had improved, and after approximately 15 weeks of treatment, dry mouth and stomatitis had abated (Yamaguchi et al., 2012).

A summary of KM for cancer treatment is presented in Table 4 (Ohara et al., 1993; Yamaguchi et al., 2002, 2011, 2012; Liao et al., 2005; Satoh et al., 2005; Matsuda et al., 2011; Takita et al., 2011; Yang et al., 2011; Ogawa et al., 2012; Misumi et al., 2013; Ikemoto et al., 2014; Okumi and Koyama, 2014).

**Table 4:** **Kampo medicine in cancer treatment**.

**Kampo medicine**	**Effects of Kampo medicine**
Chingwaysan (CWS)	Effectively treats intraoral ulcerative lesions and gingival bleeding
	Inhibits proliferation and induces apoptosis of tumor cells
	Increases Bax expression
Danshen	Inhibits OSCC proliferation
	Inhibits angiogenesis by downregulating multiple angiogenic genes
Ninjinyoeito (NYT)	Reduces nausea/vomiting and bowel movement abnormalities
	Improves motivation and fatigue/malaise
	Antioxidant effect
Rikko-san (RKS)	Pain control via local anesthesia/analgesia
	Antioxidant effect
Byakkokaninjinto (BNT)	Useful for reducing symptoms of stomatitis and xerostomia induced by radiotherapy.
Keishikajutsubuto (KJT)	Improves radiation-induced neuralgia (cold type)
Hochuekkito (HET)	Improves immune function and appetite in elderly patients
	Antioxidant effect
	Slows wasting and improves QOL.
Rikkunshito (RKT)	Contributes to the amelioration of anorectic conditions in cancer cachexia-anorexia syndrome
Orengedokuto (OGT)	Improves mucositis caused by anticancer agents
	Antioxidant effect
Juzentaihoto (JTT)	May increase long-term survival
	May induce spontaneous regression of tongue cancer
	Inhibits the progression of tumors

## Dry mouth and Sjögren's syndrome

Dryness of the mouth may occur due to reduced saliva secretion or as a sensation of dryness (thirst) in the presence of normal saliva secretion. Prescriptions are selected according to the condition of dryness (thirst or true dry mouth).

### Thirst

Patients with this condition are constantly thirsty and tend to drink copious amounts of water. The may also experience frequent thirst at night and the desire to cool the mouth with ice, either due to the presence of heat pattern (interior heat) or fluid retention. BNT, Shosaikotokakikyosekko, Makyokansekito, and KKT have heat-pattern-treating and saliva-secreting effects, while Rokumigan and Hachimijiogan are effective for thirst due to lower-region deficiency.

### Dry mouth

Other patients have dryness in the mouth but need moistening of the oral cavity rather than water. This condition is associated with dampness heat splashing sound, vital energy depression, dual deficiency of vital energy and blood, and dryness and heat due to lack of fluid. In addition to treating vital energy and fluid deficiency, dry mouth can be effectively treated with NYT and BMT, which possess heat-pattern-treating effects.

Miyazaki et al. reported that NYT improved oxybutynin hydrochloride-induced xerostomia in 12 of 16 patients diagnosed with psychogenic frequency or unstable bladder (chronic cystitis, neurogenic bladder) (Miyazaki et al., 1994).

Yamaguchi et al. reported a case of xerostomia after oral cancer treatment.

A 73-year-old female complained that lack of appetite and dry mouth following radiation therapy (40Gy), TS-1 applied at 2240 mg, and surgery for tongue cancer.

NYT 6 g/day was administered for treatment of tongue pain, dry mouth, and anorexia. Two weeks later, appetite had improved. Both dry mouth and appetite improved after 2 months of treatment (Yamaguchi et al., 2012). Yanagi et al. reported that BNT ameliorated thirst in several rat thirst models established by muscarinic receptor antagonists. This effect was associated with increased expression of aquaporin 5, suggesting that BNT enhanced salivary secretion by muscarinic (M3) receptor-mediated upregulation of aquaporin 5. In fact, this is one of several studies implicating changes in aquaporin expression in the effects of Kampo therapy for dry mouth (Yanagi et al., 2008). In contrast, Umemoto et al. reported that the muscarinic agonist cevimeline hydrochloride hydrate and histamine H2 receptor antagonist nizatidine, but not BMT, significantly increased both basal and evoked salivary secretion and relieved subjective symptoms of dry mouth (Umemoto et al., 2007). However, Nishizawa et al. found BMT was effective and safe for the relief of subjective symptoms and salivary hyposecretion associated with primary SJS in four separate randomized controlled trials (Nishizawa et al., 2002, 2003, 2004a,b). In fact, it was more effective and safer than the mucolytic bromhexine hydrochloride. Ohno reported that 27 out of 30 SJS patients in a quasi-randomized controlled trial showed increased salivary secretion following Kampo treatment (12.0 ± 1.4 ml vs. 8.2 ± 1.2 ml at baseline; *p* < 0.005) (Ohno, 2006). KM with moisturizing effects and associated studies are summarized in Table 5 (Miyazaki et al., 1994; Nishizawa et al., 2002, 2003, 2004a,b; Ohno, 2006; Umemoto et al., 2007; Yanagi et al., 2008).

**Table 5:** **Kampo medicines to treat dry mouth and/or Sjögren's syndrome**.

**Kampo medicine**	**Disease, cases**	**Study design**	**Effect**
Ninjinyoeito (NYT)	Xerostomia induced by oxybutynin (8 cases)	randomized clinical trial (RCT) Human	effective
Byakkoka ninjinto (BNT)	Increased the expression of aquaporin 5 in rat thirst models	Rat	effective
Bakumondoto (BMT)	Dry mouth (excluding Sjögren's syndrome, diabetes mellitus (DM) et al.) 100 cases	RCT Human	not effective
	Primary Sjögren's syndrome 51 cases	RCT Human	Effective
	Primary Sjögren's syndrome 115 cases (RCT)	RCT Human	Effective
	Secondary Sjögren's syndrome 424 cases (RCT)	RCT Human	Effective
	Secondary Sjögren's syndrome 380 cases (RCT)	RCT Human	Effective
	Secondary Sjögren's syndrome		
Hochuekkito (HET)	Sjögren's syndrome (28 cases)	quasi- RCT Human	Effective

### Crude drug list

Kampo medicine consists of mineral, animal, and basidiomycete as well as the crude drug of the plants.

The list of crude drug of the plants, species, family and main KM in this article were described in Table 6.

**Table 6:** **The list of crude drugs, species, family, and main kampo medicines**.

**Crude drug**	**Species and variety**	**Family**	**Main kampo medicines**
Angelicae Radix	*Angelica acutiloba* (Siebold & Zucc.) Kitagawa	Apiaceae	HET, JTT, KSS, NYT, TSS, YKS
Anemarrhenae Rhizome	*Anemarrhena asphodeloides* Bunge	Asparagaceae	BNT
Asiasari radix	*Asiasarum sieboldii* F. Maekawa	Aristolochiaceae	RKS
	*Asarum sieboldii* Miq.		
Astragali Radix	*Astragalus membranaceus* Bunge	Leguminosae	HET, JTT, NYT
	*Astragalus mongholicus* Bunge, Mém. Acad. Imp. Sci. Saint Pétersbourg		
Atractylodis Lanceae Rhizoma	*Atractylodes lancea De* Candolle.	Asteraceae	HET, JTT, KJT, KSS, NYT, RKT, TSS, YKS
	*Atractylodes lancea* (Thunb.) DC.		
	*Atractylodes japonica* Koidzumi ex Kitamura		
Aurantii nobilis Pericarpium	*Citrus deliciosa* Ten	Rutaceae	HET, NYT, RKT
Bupleuri Radix	*Bupleurum falcatum* Linné	Apiaceae	HET, KSS, SBT, SRB, SST, YKS
Cimicifugae Rhizoma	*Actaea simplex* (DC.) Wormsk. ex Prantl	Ranunculaceae	HET, RKS
Cinnamomi Cortex	*Cinnamomum cassia* (L.) J.Presl	Lauraceae	JTT, KJT, NYT, ORT, SRB
	*Cinnamomum cassia* (L.) J.Presl in F.Berchtold and J.S.Presl		
Cnidii Rhizoma	*Cnidium officinale* Makino	Apiaceae	JTT, TSS, YKS
	*Ligusticum officinale (Makino) Kitag*		
Gardeniae Fructus	*Gardenia jasminoides* J.Ellis	Rubiaceae	KSS, OGT
Gentianae scabrae Radix	*Gentiana scabra* Bunge	Gentianaceae	RKS
Ginseng Radix	*Panax ginseng* C. A. Meyer	Araliaceae	BMT, BNT, HET, HST, JTT NYT, ORT, RKT, SBT, SRB, SST, YKS
Magnoliae Cortex	*Magnolia obovate* Thunberg	Magnoliaceae	SBT
	*Magnolia officinalis* Rehder and E.H.Wilson in C.S.Sargent		
Menthae Herba	*Mentha canadensis* Linné	Lamiaceae	KSS
Moutan Cortex	*Paeonia suffruticosa* Andrews	Paeoniaceae	KSS
Ophiopogonis Tuber	*Ophiopogon japonicus* (Thunb.) Ker Gawl.	Asparagaceae	BMT
Oryzae Fructus	*Oryza sativa* Linné	Poaceae	BMT, BNT
Paeoniae Radix	*Paeonia suffruticosa* Andrews	Paeoniaceae	JTT, KJT, KSS, NYT, TSS
	*Paeonia lactiflora* Pallas		
Perillae Herba	*Perilla frutescens* (L.) Britton	Lamiaceae	SBT
	*Perilla frutescens* var. crispa (Thunb.) H.Deane		
Phellodendri Cortex	*Phellodendron amurense* Ruprecht	Rutaceae	OGT
	*Phellodendron chinense* C.K.Schneid.		
Pinelliae Tuber	*Pinellia ternata* (Thunb.) Makino	Araceae	BMT, HST, ORT, RKT, SBT, SRB, SST
Platycodi Radix	*Platycodon grandiflorum* A. De Candolle	Campanulaceae	KKT
Processi Aconiti Radix	*Aconitum japonicum* Thunberg	Ranunculaceae	KJT
	*Aconitum carmichaelii* Debeaux		
	*Aconitum japonicum* Thunb. *in* J.A.Murray		
Rhemanniae Radix	*Rehmannia glutinosa* (Gaertn.) DC.	Plantaginaceae	JTT, NYT
Saposhnikoviae Radix	*Saposhnikovia divaricata* Schischkin	Apiaceae	RKS
	*Saposhnikovia divaricata* (Turcz.) Schischk. in V.L.Komarov (ed.)		
Uncariae Uncis cum Ramulus (Uncaria Hook)	*Uncaria rhynchophylla* (Miq.) Miq. ex Havil.	Rubiaceae	YKS
	*Uncaria sinensis* (Oliv.) Havil., J. Linn. Soc.		
	*Uncaria macrophylla* Wall. in W. Roxburgh		
Zingiberis Rhizoma	*Zingiber officinale* Roscoe	Zingiberaceae	HET, HST, KJT, KSS, ORT, RKT, SBT, SRB, SST
Zizyphi Fructus (Jujube)	*Zizyphus jujuba* Miller var. *inermis* Rehder	Rhamnaceae	BMT, HET, HST, KJT, ORT, RKT, SBT, SRB, SST
	*Ziziphus jujuba* var. *inermis* (Bunge) Rehder		

http://www.theplantlist.org/; http://mpns.kew.org/mpns-portal/

The crude drugs were described in a lot of articles and species were described in only three articles in this paper (Dragland et al., 2003; Yanagi et al., 2008; Suzuki et al., 2012).

This search was carried out using “http://www.theplantlist.org/, and http://mpns.kew.org/mpns-portal/”.

The crude drugs of mineral, animal and basidiomycota were cited from “http://jpdb.nihs.go.jp/jp16e/” (The Japanese Pharmacopoeia Sixteenth Edition, 2011) and

“http://wakankensaku.inm.u-toyama.ac.jp/wiki/Persist:CrudeDrugList”.

The list of crude drug of mineral, animal and basidiomycota, scientific names, family and main KM in this article were described in Table 7.

**Table 7:** **The list of Crude drugs of Mineral, Animal, Basidiomycota, and kampo medicines**.

**Crude drugs of mineral, animal, and Basidiomycota**			
**Crude drug**	**Scientific name**	**Family**	**Kampo medicines**
Fossilia Ossis Mastodi (Longgu)	Mineral	(−)	SRB
Gypsum Fibrosum Gypsum	Mineral	(−)	BNT
Ostreae Testa (Oyster Shell)	*Ostrea gigas* Thunberg	Osteridae	SRB
Poria Sclerotium	*Wolfiporia cocos* Ryvarden et Gilberson	Polyporaceae	JTT, KSS, NYT, RKT, SBT, SRB, TSS, YKS
	*Poria cocos* Wolf		

http://jpdb.nihs.go.jp/jp16e/; http://wakankensaku.inm.u-toyama.ac.jp/wiki/Persist:CrudeDrugList

## Discussion

The human oral cavity is the initial digestive organ and is likely to be affected by various local stimuli and microorganisms. And the oral cavity is an anatomically complex structure that has evolved to perform a multitude of functions

Therefore, it is necessary to use Kampo theory, such as Ki (vital energy), Ketsu (blood), and Sui (fluid) abnormalities, to treat oral diseases.

(1) In Kampo medicine, the treatment of stomatitis differs depending on whether the symptoms are intense acute or chronic. In acute stomatitis, heat because of agitated vital energy may affect the head, chest, and middle abdominal region. Therefore, treatment is required to cool the heat in the heart, stomach, and liver using formulae with cooling effects. HST and ORT cool the digestive organ. OGT calms irritation and cools whole-body fever, and BNT controls physical fever and thirst and provides moisture to the body.

Stomatitis is also related to the generation of ROS; therefore, the antioxidant and anti-inflammatory activities of medicinal herbs can be effective (Oka, 1995; Dragland et al., 2003; Horie et al., 2008; Yamaguchi et al., 2008; Kono et al., 2010; Li et al., 2010; Zheng et al., 2011; Aoyama et al., 2014). There are numerous antioxidants in crude KM (Dragland et al., 2003) (Table 1). HST is useful for stomatitis medicamentosa and radiostomatitis (Kono et al., 2010; Aoyama et al., 2014). Thus, we can control environmental factors (cold, heat, dampness, dryness) and vital energy, blood, and fluid of the organ systemically to treat stomatitis as well as reduce causative ROS.

(2) BMS, glossalgia, and AFP are chronic pain conditions with no clear underlying pathology. Furthermore, these are multifactorial syndromes involving the interaction of biological and psychological factors (Baker and Savage, 2005; Klasser et al., 2008, 2011; Ferensztajn et al., 2013; Yamaguchi, 2013). Nonsteroid anti-inflammatory drugs (NSAIDs) are effective for acute pain but are not very effective for chronic pain. The balance of environmental factors (cold, heat, dampness, and dryness) differs between acute and chronic pain (Yamaguchi, 2013). Local temperature decrease and edema often occur in chronic pain. These conditions result from local circulatory disturbances and can be resolved by improving the flow of blood and fluid. Local temperature decrease and edema are closely associated with the balance of Ki (vital energy), Ketsu (blood), and Sui (fluid) (Yamaguchi, 2013).

Glossalgia in patients with a deficiency pattern associated with vital energy depression can be treated with HET, which upregulates the microcirculation.

TSS not only controls the balance of blood and fluid but also improves the peripheral circulation (Takayama et al., 2014). KSS is used for nonspecific complaints and upper heat/lower cold in deficiency-pattern patients. Both TSS and KSS improve microcirculation and autonomic nerve imbalance.

Several reports investigated the contribution of the capsaicin receptor TRPV1 (Gear et al., 1999; Yilmaz et al., 2007), which mediates heat-like oral pain and NGF in BMS. RKS contains crude Saisin, which has a chemical constitution resembles capsaicin. RKS is thought to be effective against BMS caused by the peripheral TRPV1 receptor.

Several KM, such as SRB (Mizoguchi et al., 2003), YKS (Mizoguchi et al., 2010; Suzuki et al., 2012), KSS, and SBT (Bessho et al., 1998), can reduce stress and associated pain by altering glutamatergic and monoaminergic transmission in the brain. These formulae are broadly effective treatments for BMS, glossalgia, atypical maxillofacial pain, and atypical dental pain.

The clinical efficacy of KM for BMS and AFP may depend on the regulation of the mesolimbic dopaminergic and descending glutamatergic pain modulation systems. Moreover, these agents appear to suppress the psychological sequel of depression and anxiety (Tables 2, 3).

### Oral cancer

A deficiency of Ki, Ketsu, and Sui are present in oral cancer patients. Kampo therapy is usually used as the adjuvant therapy, side effect reduction and improvement of the overall status and/or oral discomfort.

1. Inhibition of the proliferation of cancer cells: CWS effectively treats intraoral ulcerative lesions and gingival bleeding, inhibits tumor cell proliferation, and induces tumor cell apoptosis (Liao et al., 2005).
2. Adjuvant therapy of the main cancer therapy: HET increased lymphocyte cell-surface antigens, CD3-positive cells, and CD3/CD4 double-positive cells in elderly patients (Satoh et al., 2005). Administration of JTT significantly decreased Treg populations and increased the CD4/CD8 ratio (Ikemoto et al., 2014).
3. Side effect reduction of main anti-cancer therapy:Kampo medicine is effective for symptom improvement during oral cancer treatment, such as digestive symptoms, impaired hematopoiesis, general malaise, and stomatitis medicamentosa. Hozai, such as RKT, HET, JTT, and NYT, content the crude drugs of the treatment of digestive symptoms and blood formation. RKT and HET include the effective crude drags of anorexia such as Atractylodis Lanceae Rhizoma, Ginseng Radix, Zizyohi Fructus, and Glycyrrhizae Radix. Also JJT and NYT include the effective crude drags of anorexia such as Atractylodis (Lanceae) Rhizoma, Ginseng Radix, Poria (Holelen) and Glycyrrhizae Radix. Therefore, these KM are used for anorexia and general malaise.
  Furthermore, JJT include the crude drugs (Angelicae Radix, Paeoniae Radix, Cnidii Rhizoma, and Rehmanniae Radix) with the hematopoiesis. NYT include Angelicae Radix, Paeoniae Radix, and Rehmanniae Radix. Therefore, these KM are used for anemia and leukocytopenia as an adjuvant therapy.
  HET, JTT, and NYT also improve immune function (Satoh et al., 2005; Takita et al., 2011; Ikemoto et al., 2014), digestive symptoms, and general malaise in cancer patients (Misumi et al., 2013; Okumi and Koyama, 2014).
4. Improvement of quality of life such as the overall status and/or oral discomfort:
  Those Hozai such as HET, JTT and NYT improve Ki, Ketsu, and Sui deficiencies in cancer patients with palliative care. Kampo formulae are useful for reducing the unpleasant side effects of cancer therapy (Table 4).
  (4) Heat- and cold-dryness stages exist in both dry mouth and Sjögren's syndromes.
  The heat-dryness stage belongs to Yo and cold-dryness stage belongs to Yin. BNT is useful for the heat-dryness stage (Yo) (Yanagi et al., 2008) and NYT (Miyazaki et al., 1994), BMT (Umemoto et al., 2007; Nishizawa et al., 2002, 2003, 2004a,b) and HET (Ohno, 2006) possess moisturizing effects in the cold-dryness stage (Yin) (Table 5). In addition, BMT and GRS affect aquaporin five expression, and thereby improve salivation (Tsubota et al., 2001).

## Conclusion

This article examined 54 studies and summarized pharmacological actions and clinical applications of Japanese herbal medicines in various refractory oral diseases such as stomatitis, BMS, glossalgia, AFP, oral cancer, dry mouth, and SJS from the standpoint of Kampo theory.

These analyses are summarized in the Tables 1–5, and the list of crude drugs, species, family, and main KM in this article were described in Tables 6, 7.

Kampo therapy is useful for the treatment of oral diseases that cannot be cured effectively by western medicine.

### Conflict of interest statement

The author declares that the research was conducted in the absence of any commercial or financial relationships that could be construed as a potential conflict of interest.
